# 

**Published:** 1887-07

**Authors:** 


### TABLE OF CONTENTS.

### Original and Selected Articles.

I. Amyloid Degeneration of the Kidney.—II. Endo-Cardial and Peri-Cardial Murmurs, by James Tyson, M.D., Philadelphia, Pa .......... 265
Secondary Syphilis of Middle Ear; Unilateral Facial Paralysis, by S. Latimer Phillips, M. D., Savannah, Ga .......... 268
Snakes, Snakes .......... 270
The Operation of Cæsarean Section Performed on a Cow, by C. Hamilton, M.D., Ringgold, Georgia .......... 272
A Case of Maternal Impression, by S. W. Stiles, M.D., Georgia .......... 273
The New Theory of Yellow Fever Inoculation.. 274
Hemorrhagic Malarial Fever, by Benj. H. Riggs, M.D., Selma, Alabama .......... 277

### Abstracts and Gleanings.

Placenta Prævia, with Uterine Hemorrhage... 280
Charcoal and Camphor .......... 281
Suppurative Inflammation of the Middle Ear 282
The "Rational Method" of After-treatment of Cataract Operations, Iridectomies, etc .......... 283
Cancers—Orders and Treatment .......... 285
Sciatica Treated by Galvanism .......... 286
Distilled Water in Eye Lotions .......... 287
Treatment of Acute Intestinal Obstruction; Hypnone; Sarracenia Flava in Sporadic Dysentery .......... 288
Berberis Aquifolium, Rhus Aromatica; Cocaine Inebriety .......... 289
Phosphorus in Intermittent Fever; Iodide of Potassium in Diphtheria; Belladonna in Sterility of Females .......... 290
Penicious Hemorrhagic Malarial Fever; Posology and Use of Some New Remedies...... 291
Ague Treated by Quinine and Opium; Photographing the Uterine Cavity .......... 292
Thapsia Plaster; Elixir Iron Pyrophosphate, Quinine and Strychnine; Linseed Oil as a Remedy for Itching; Toothache Drops..... ... 293

### Scientific Items.

The Living Earth; A Field of Work under the Sea .......... 294
Simple Test for Wall Paper; Detection of Micro Organisms .......... 295

### Practical Notes and Formulæ.

Bloody Flux; Diabetis Mellitus..........296
For Dysentery; Camphor Vapor in Bronchitis; The Treatment for Colds .......... 297
Treatment of Stye; Constipation of Children; Ointment for the Relief of Ocular Pains; Glycerin in Phthisis .......... 298

### Editorials and Miscellaneous.

To Our Subscribers; Editorial Brevities; Consumption Treated by Gaseous Enemata ...... 299
A Needle in the Arm—How Did it Get There; Ingratitude .......... 300
The American Medical Association .......... 302
Books and Pamphlets Received .......... 306
Receipted; Special Notes .......... 307

